# Preparation of ZIF@ADH/NAD-MSN/LDH Core Shell Nanocomposites for the Enhancement of Coenzyme Catalyzed Double Enzyme Cascade

**DOI:** 10.3390/nano11092171

**Published:** 2021-08-25

**Authors:** Le Wang, Pengxue Sun, Yiyu Yang, Hanzhen Qiao, Hailong Tian, Dapeng Wu, Shuoye Yang, Qipeng Yuan, Jinshui Wang

**Affiliations:** 1 College of Biological Engineering, National Engineering Laboratory for Wheat & Corn Further Processing, Henan University of Technology, Zhengzhou 450001, China; lewang@mail.haut.edu.cn (L.W.); pengxuesun@mail.haut.edu.cn (P.S.); yiyuyang@mail.haut.edu.cn (Y.Y.); hanzhenqiao@mail.haut.edu.cn (H.Q.); hailongtian@mail.haut.edu.cn (H.T.); shuoyeyang@mail.haut.edu.cn (S.Y.); 2 School of Environment, Henan Normal University, Xinxiang 453001, China; 3 State Key Laboratory of Chemical Resource Engineering, Beijing University of Chemical Technology, Beijing 100029, China

**Keywords:** confinement effect, multi-enzyme catalytic cascade, in-situ synthesis, coenzyme recycling, co-immobilization enzyme

## Abstract

The field of enzyme cascades in limited microscale or nanoscale environments has undergone a quick growth and attracted increasing interests in the field of rapid development of systems chemistry. In this study, alcohol dehydrogenase (ADH), lactate dehydrogenase (LDH), and mesoporous silica nanoparticles (MSN) immobilized nicotinamide adenine dinucleotide (NAD^+^) were successfully immobilized on the zeolitic imidazolate frameworks (ZIFs). This immobilized product was named ZIF@ADH/NAD-MSN/LDH, and the effect of the multi-enzyme cascade was studied by measuring the catalytic synthesis of lactic acid. The loading efficiency of the enzyme in the in-situ co-immobilization method reached 92.65%. The synthesis rate of lactic acid was increased to 70.10%, which was about 2.82 times that of the free enzyme under the optimal conditions (40 °C, pH = 8). Additionally, ZIF@ADH/NAD-MSN/LDH had experimental stability (71.67% relative activity after four experiments) and storage stability (93.45% relative activity after three weeks of storage at 4 °C; 76.89% relative activity after incubation in acetonitrile-aqueous solution for 1 h; 27.42% relative activity after incubation in 15% N, N-Dimethylformamide (DMF) solution for 1 h). In summary, in this paper, the cyclic regeneration of coenzymes was achieved, and the reaction efficiency of the multi-enzyme biocatalytic cascade was improved due to the reduction of substrate diffusion.

## 1. Introduction

The coenzyme regeneration is to regenerate the coenzyme from the oxidized state to the reduced state, or vice versa, so as to keep the coenzyme at a certain level of catalyst amount. In recent years, in order to solve the problem of coenzyme regeneration, a series of methods have been proposed, including regeneration systems such as chemistry, photochemistry, enzymology, and electrochemistry [1,2,3,4].

The multi-enzyme cascade reaction referred to the description that the product generated through efficient transfer of the intermediates from one catalytic site to another is achieved by the formation of enzyme complexes, and then reaction will continue until its completion [5,6,7]. The addition of various enzymes in a defined three-dimensional space to initiate multi-enzyme cascade reaction can reduce the transport time of the substrate and intermediate loss due to diffusion, thus improving the efficiency of the biocatalytic cascade compared with the non-tissue diffusion-controlled biocatalysts environment [8,9]. Two-enzyme and three-enzyme cascade reactions have been reported—such as the spatial localization of enzymes on DNA scaffolds [10], DNA bands [11], DNA chains [12], or DNA folding [13]—have been used to operate two or three enzyme cascades. 

In the past few decades, multi-enzyme immobilization methods have continued to emerge. The methods that have been reported include multi-site covalent binding [14], co-embedding [15], co-adsorption [16], and specific binding [17] to immobilize multiple enzymes. Compared with the free enzyme, the intermediate product of the reaction in the multi-enzyme complex can be quickly transferred to reduce the diffusion loss, the local concentration of the intermediate product is increased, and the catalytic efficiency of the enzyme is improved [18]. Therefore, the study on the reliability strategy of multi-enzyme catalytic cascade and encapsulation of multi-enzyme plays an important role in enzyme homogeneous catalysis [19,20]. 

In addition, after combining these cascade multi enzymes with solid carriers, they can be recovered from the action mixture in a simple way. The repeatability of the immobilized enzyme is beneficial to reduce the cost. Also, the immobilization of enzyme can improve the stability of temperature and pH value. Torabi [21] covalently immobilized cholesterol oxidase (COD) and horseradish peroxidase (POD) on the surface of perlite. The pH stability of the immobilized double enzyme was higher than that of the free enzyme. The recovery rate of the enzyme activity of the co-immobilized multi-enzyme was 65% after repeated use for 20 times, which indicated that the immobilized enzyme had a good reuse rate. Talekar [14] used glutaraldehyde to crosslink α-amylase, glucoamylase, and pullulanase to prepare co-crosslinked enzyme polymers. The stability of the immobilized multiple enzymes was greatly improved. In addition, the performance of the immobilized multiple enzymes had not changed significantly after being reused five times. Therefore, the co-immobilization of multi-enzymes with cascade reaction had good application potential [22,23,24].

At present, studies on the encapsulation and immobilization of individual enzymes have been done relatively well, including the stability and catalytic activity of the encapsulated enzyme, catalyzing redox reactions during metabolism and the conversion efficiency of the substrates [25,26,27,28]. While there are still many deficiencies in the immobilization of multi-enzymes and the cascade action between multi-enzymes. Especially when multiple enzymes are immobilized or encapsulated, their active sites could be affected by multiple factors, such as the reaction conditions [29], the type of immobilized carrier, the way of enzyme immobilization [30], the microenvironment surrounding the enzyme molecule and so on [31]. In addition, when choosing the co-immobilized carrier, it was more important that the carrier should introduce a suitable concomitant microenvironment for the biocatalyst after enzyme incorporation. Moreover, they should have a reasonable diffusion rate for substrates and products. Enzyme immobilization materials should also have good structural stability, and be able to maintain the original structure after repeated experiments to avoid leakage of the enzyme [22].

Metal organic framework (MOFs) are used in multi-enzyme cascade reactions due to their excellent properties [32,33]. Zeolitic imidazolate framework-8 (ZIF-8) is a representative material in the metal framework compounds series with rhombic dodecahedral structure [34]. ZIF-8 was used to encapsulate multi-enzymes in the form of in-situ coprecipitation polymerization of Zn^2+^ metal ions and 2-methylimidazole organic ligands in aqueous phase. This method of immobilization is mild and can be completed at room temperature, which can avoid the folding damage to the enzyme structure of a great extent. Therefore, ZIF-8 can be used as the material of choice for immobilized enzymes.

Research on enzymatic regeneration of coenzymes is mainly focused on finding a suitable regeneration system. The currently used regeneration enzymes include: formate dehydrogenase (FDH), glucose dehydrogenase (GDH), alcohol dehydrogenase, and hydrogenase. ADH and LDH were used as the two key enzymes for coenzyme regeneration The catalysis of ethanol by ADH initiated the conversion of NAD^+^ to NADH. Pyruvate could regenerate NAD^+^ in the process of catalyzing the synthesis of lactic acid with the participation of NADH. Also, the MSN was selected as the immobilized carrier of NAD^+^. Under the action of the accelerator carbodiimide (EDC), the amide bond was formed by the inactive site -NH of NAD^+^ and -COOH modified on the surface of MSN [35]. This study prepared core–shell nanocomposites in two steps based on the coenzyme regeneration strategy and substrate channeling principle. A stable MSN-NAD was designed and synthesized in the first step. The second step was the immobilization and in-situ embedding of LDH, MSN-NAD, and ADH with ZIF-8. In order to improve the efficiency of multi enzyme cascade and mass transfer, the multi enzyme system was limited in the nanostructured reaction region of ZIF-8 to reduce the diffusion of solvent. The NAD^+^/NADH functionalized polymer studied in this project was limited in the shell of ZIF-8. In this way, the conversion of pyruvate by LDH could be accelerated with the increasing of lactic acid yield. Moreover, the application of coenzyme regeneration technology could reduce the addition of expensive coenzymes in the reaction system and reduces the reaction cost.

## 2. Materials and Methods

### 2.1. Chemicals and Reagents

LDH (Sigma–Aldrich, St. Louis, MO, USA), Bovine albumin (Sigma–Aldrich, St. Louis, MO, USA), ADH (Sigma–Aldrich, St. Louis, MO, USA), NAD^+^ (RUIBIO, ≥98%), Pyruvic acid (98%), 3-Aminopropyltriethoxysilane (APTES, 98%), 1-(3-Dimethylaminopropyl)-3-ethylcarbodiimide hydrochloride (EDC·HCL, >99.0%) were purchased from Hefei Bomei biological Company (Hefei, China). Tetraethyl orthosilicate (TEOS, 98%), cetyltrimethylammonium bromide (CTAB, >99%), diethanolamine (DEA, 99%), zincacetate (Zn(CH_3_COO)_2_, 99.99%), and 2-methylimidazole (98%) were purchased from Aladdin Chemistry Co., Ltd. (Shanghai, China). All other reagents were purchased from Tianjin Weiyi Chemical Technology Co., Ltd. (Tianjin, China).

### 2.2. Immobilized Coenzyme

The MSN was synthesized according to literature methods [36,37] and was modified by amino group according to the following method. 200 mL of C_2_H_5_OH and 700 mL of APTSES were added to 0.2 g MSN. The reaction was controlled to reflux and stir at 70–80 °C for 24 h. After the reaction, the product was washed with 80% methanol solution and deionized water and then lyophilized and recovered. The purpose of modifying the amino group on the surface of the amino group was to modify the carboxyl group. 2 g Carboxyl modified mesoporous silica nanoparticles (MSN-COOH) were dispersed in 30 mL of deionized water and sonicated for 10 min. Then added a certain amount of NAD^+^ under magnetic stirring. 50 mg water-soluble carbodiimide EDC was continuously added to the solution within 5 min. During this process, the pH was adjusted to 4.8 with 0.5 M hydrochloric acid solution. The reaction temperature and time were 28 °C and 28 h. After the reaction, the unreacted NAD^+^ and other impurities were removed by centrifugation, and then washed with 0.2 M NaCl solution until none of NAD^+^ was detected in the washing solution. Finally, the immobilized coenzyme NAD^+^ was obtained by freeze-drying. The process of immobilization of coenzyme is shown in Figure S1.

### 2.3. Preparation of ZIF@ADH/NAD-Polymer/LDH 

1 mg ADH (300 U/mg), 1 mg LDH (250 U/mg) and 2 mg MSN-NAD^+^ were added to 10 mL zinc acetate (40 mM) solution. The 10 mL (2.8 mM) 2-methylimidazole was quickly added at room temperature and stirred overnight. The mixture was precipitated and centrifuged. The solid content was washed with deionized water for several times, and then freeze-dried to obtain ZIF@ADH/NAD-MSN/LDH. 

### 2.4. Materials Characterization

The samples were measured by Fourier transforms infrared spectroscopy (FTIR, Thermo Scientific, Nicolet IS20, Waltham, MA, USA), scanning electron microscopy (SEM, HITACHI, S4800, Tokyo, Japan) and Transmission electron microscopy (TEM, HITACHI, HT7700, Tokyo, Japan). Rhodamine B and fluorescein isothiocyanate (FITC) were used to label LDH and ADH respectively. The Confocal laser scanning microscope (CLSM, Olympus, FV3000, Tokyo, Japan) was used to characterize the distribution of the two enzymes in the polymer system. The changes of specific surface area and porosity before and after immobilization of enzyme were studied by specific surface area analyzer (BET, bjbuilder, SSA, Beijing, China).

### 2.5. NAD^+^-Mediated Enzyme-Linked Reaction

100 μg/mL ZIF@ADH/NAD-MSN/LDH, 2 mM pyruvate, 20 mM ethanol were added to 200 μL PB buffer solution (100 mM, pH 7.4), respectively. Equal amounts of ADH, MSN-NAD^+^, and LDH were used as controls. The reaction was performed for 3 h, and the concentration of lactic acid was measured. The effect of the cofactor-enzyme cascade was evaluated by detecting the rate of lactic acid produced. 

The concentration of lactic acid was measured with HPLC. The specific method of HPLC was as follows. Column: C18 4.6 × 150 mm, 5 μm. Mobile phase: 2.5% ammonium dihydrogen phosphate adjusted pH 2.65 with phosphoric acid. Flow rate: 1.0 mL/min. Injection volume: 5 μL. Detection wavelength: 210 nm.

### 2.6. Determination of Protein Concentration and Enzyme Activity 

A series of concentration gradient BSA standards were prepared. After adding 5 mL Brad ford reagent and reacting for 3 min, the absorbance of the solution at the beginning of 595 nm was measured. The standard curve was drawn with the absorbance at BSA concentration 595 nm [38]. The enzyme loading efficiency of the carrier could be calculated by measuring the total protein content in the supernatant and washing fluid. The enzyme loading efficiency (E) was calculated by the Formula (1).

(1) $$E\%=\left[\left(m_{0}-m_{1}\right)/m_{0}\right]\times 100$$

Pyruvate can react with 2,4-dinitrophenylhydrazine to produce 2,4-dinitrophenylhydrazone (reddish brown, alkaline conditions). A spectrophotometer was used to detect the absorbance of the reaction solution at 520 nm to analyze the content of pyruvate. At 37 °C, the pyruvate consumption of 1 nmol per milligram of protein per minute was defined as 1 U (the default was that the load of ADH and LDH was the same in @ADH/NAD-polymer/LDH). The ratio of enzyme activity before and after immobilization was used to evaluate the effect of immobilization on enzyme activity.

### 2.7. Stability of ZIF@ADH/NAD-MSN/LDH

#### 2.7.1. Stability Experiment of Temperature and pH for ZIF@ADH/NAD-MSN/LDH

The temperatures of 30, 35, 40, 45, and 50 °C were investigated the effect of the lactic acid production rate. Preparation of ZIF@ADH/NAD- Polymer/LDH was consistent with Section 2.3. Similarly, the pH of 6.5, 7, 7.5, 8, 8.5, and 9 were investigated the effect of the lactic acid production rate.

#### 2.7.2. Storage and Experimental Stability of ZIF@ADH/NAD-MSN/LDH

ZIF@ADH/NAD-MSN/LDH was washed and centrifuged with PBS solution for recovery. The recovered polymer is re-applied to the catalytic cascade of lactic acid. After five repeated uses, the production rate of lactic acid was tested. The relative activity was calculated by the following Formula (2).

(2) $$\mathrm{Relative}\;\mathrm{activity}\;\left(\%\right)=(\mathrm{Lactic}\;\mathrm{acid}\;\mathrm{production}\;\mathrm{rate}/\;\mathrm{Initial}\;\mathrm{Lacticacid}\;\;\mathrm{production}\;\mathrm{rate})\times 100$$

ZIF@ADH/ NAD-polymer /LDH and free enzymes were stored in a refrigerator at 4 °C for 3 weeks, respectively. The initial enzyme activity was defined as 100%. The relationship between enzyme activity and storage time before and after immobilization was investigated.

#### 2.7.3. Stability of Organic Reagents

After incubating ZIF@ADH/NAD-MSN/LDH, MSN-NAD^+^, LDH, ADH and NAD^+^, LDH, ADH in 15% DMF and 15% acetonitrile solution for 1 h, their activities were determined by detecting the production rate of lactic acid, and the formula for calculating the relative activity of the enzyme was the same as that of Section 2.7.2.

### 2.8. Statistical Analysis

The data was processed by Origin 8.5. ANOVO test was performed by SPSS 22.0 data processing system, and Duncan’s new multiple range method was used for multiple comparison analysis. All experiments were performed in triplicates, and statistical significance was represented by *p* values [39].

## 3. Results and Discussion

### 3.1. Morphological and Textural Properties of ZIF@ADH/NAD-MSN/LDH

**Figure 1:**
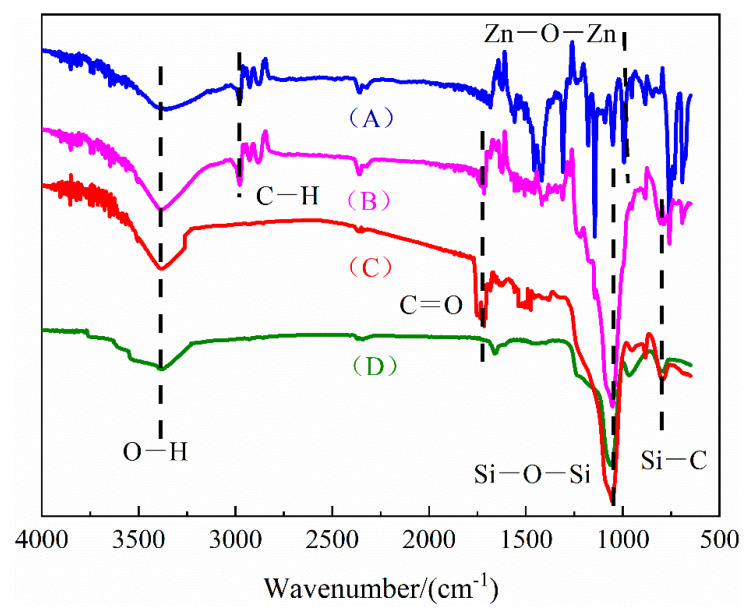
FT-IR spectra in range of 4000–500 cm^−1^. (**A**) Zeolitic imidazolate framework-8 (ZIF-8, blue line); (**B**) Core–shell nanocomposite prepared by ZIF-8 immobilized ADH, LDH, and MSN-NAD (ZIF@ADH/NAD-MSN/LDH, purple line); (**C**) Carboxyl modified silica nanoparticles (MSN-COOH, red line); (**D**) Mesoporous silica nanoparticles (MSN, green line).

FT-IR spectra of ZIF-8 before and after loading with enzyme are shown in Figure 1 (line A,B). The peaks center of 1580, 1150, and 994 cm^–1^ indicated the stretching of C–N bond in the ZIF-8 [40]. The 2933 cm^–1^ was the stretching vibration of the C–H bond at the imidazole ring. None of the hydrogen bond (N–H···N) absorption peaks and the vibration absorption peak of the N–H bond in the imidazole ring were found at 2600 cm^−1^ and 1843 cm^–1^, indicating that 2-methylimidazole was completely deprotonated. The adsorption band appeared at 1090 cm^−1^ was attributed to Zn–O–Zn from ZIF@ADH/NAD-MSN/LDH [41].

FT-IR spectra of MSN before and after amino modification are shown in Figure 1 (line C,D). The characteristic peak at 1100 cm^–1^ was attributed to Si–O–Si asymmetric stretching vibrations. The characteristic absorption peak of Si–C is located at 890 cm^–1^ [42]. The adsorption band appeared at 3300 and 1720 cm^–1^ are attributed to the O–H and C=O from MSN–COOH [43]. It indicated the modification of the carboxyl group on the surface of MSN. In summary, it indicated the preparation of the ZIF@ADH/NAD-MSN/LDH conformed to experimental expectations.

**Figure 2:**
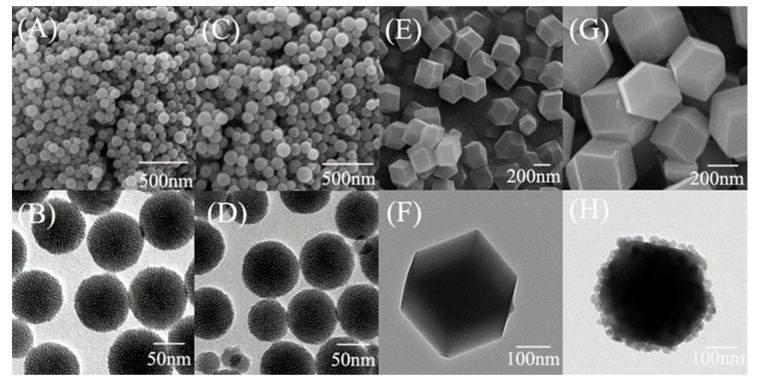
Scanning electron microscope (SEM) and Transmission electron microscopy (TEM) images of different materials. (**A**) SEM of mesoporous silica nanoparticles (MSN), (**B**) TEM of MSN, (**C**) SEM of carboxyl modified silica nanoparticles (MSN-COOH) (**D**) TEM of MSN-COOH, (**E**) SEM of zeolitic imidazolate framework-8 (ZIF-8) (**F**) TEM of ZIF-8, (**G**) SEM of core–shell nanocomposite prepared by ZIF-8 immobilized ADH, LDH and MSN-NAD (ZIF@ADH/NAD-MSN/LDH), (**H**) TEM of ZIF@ADH/NAD-MSN/LDH.

The images of SEM and TEM are shown in Figure 2. The SEM and TEM images of MSN are shown in Figure 2A,B. The SEM and TEM images of MSN-COOH are shown in Figure 2C,D. It can be seen that the particle size was little different between the MSN before and after the amino modification. MSN was a spherical structure with a size of 70–90 nm. The surface of MSN-COOH and MSN showed uniformly distributed light-colored dark spots, which represented the synthesis of porous MSN-NH_2_ with uniform pores. As can be seen from Figure 2 and Figure S2, MSN was a material with the rich mesoporous structure. The existence of mesopores and macropores could highly accelerate the mass transfer of reactants diffusing into and reacted products going out of the MOF catalyst [44]. Therefore, it could be deduced that the MSN with the rich mesoporous structure was beneficial to the diffusion rate of the substrate.

From the Figure 2E–G, it showed that ZIF-8 and ZIF@ADH/NAD-MSN/LDH had the same rhombic dodecahedral structure, which was agreement with the results in the literatures [34,45]. It was concluded that the loading of MSN-NAD and enzyme had less effect on the crystal synthesis of ZIF-8 [34,46,47]. Compared with the clear and transparent ZIF-8 material in Figure 2F, the inside of the material in Figure 2H was fuzzy and opaque, indicating that there were other substances encapsulated inside. The particle size of ZIF@ADH/NAD-MSN/LDH was about 400–500 nm. The interaction between MSN surfaces carboxyl groups and Zn^2+^ was essential for inducing the growth of ZIF-8 on the MSN-COOH surface and constructing a uniform core–shell nanostructure [48,49]. Also, there were smaller spherical particles around ZIF@ADH/NAD-MSN/LDH. By analyzing the size of these particles, these may be the MSN particles that were not wrapped by ZIF-8 [50].

**Figure 3:**
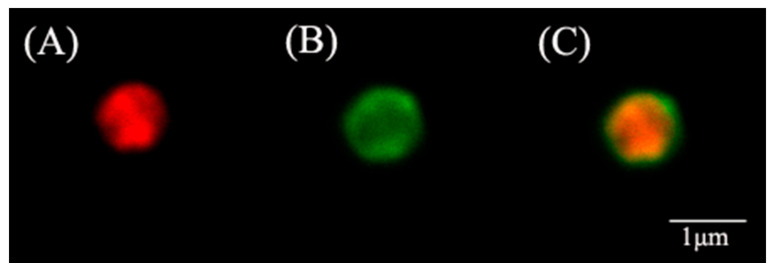
Images of confocal laser scanning microscope. (**A**) Distribution of LDH labeled with Rhodamine B; (**B**) Distribution of ADH labeled with fluorescein isothiocyanate (FITC); (**C**) Distribution of two enzymes in core–shell nanocomposites prepared by ZIF-8 immobilized ADH, LDH, and MSN-NAD (ZIF@ADH/NAD-MSN/LDH).

After fitting, the equation of bovine serum protein concentration and absorbance was Y = 0.938X + 0.026, R^2^ = 0.9968. The total immobilization efficiency (E) of the two enzymes was calculated to be 92.65%, and the immobilization amount of the two enzymes was 40 μg/mg. CLSM about ZIF@ADH/NAD-MSN/LDH is shown in Figure 3. The red fluorescence excited at 543 nm represents that ADH was encapsulated in the ZIF-8 structure (Figure 3A). Similarly, the green fluorescence excited at 488 nm represents that LDH was encapsulated (Figure 3B). From Figure 3A,B, it indicated that the two enzymes were confined to the ZIF-8 carriers rather than attached to the surface (Figure 3C). This may be related to the coordination between Zn^2+^ and protein amide bond [51,52]. The amide bond facilitated the enzyme immobilization during the crystal synthesis process of ZIF-8 [53]. The laser confocal map showed the limiting effect of the material on the enzyme, which was also corresponding to the results of electron microscope in Figure 2. The restriction effect of materials on enzymes could reduce the mass transfers efficiency of multiple enzymes in the environment and increase the conversion rate of coenzymes [54]. Therefore, the conversion to ethanol and pyruvate could be completed quickly.

The specific surface area and pore structure of ZIF-8, MSN, MSN-COOH, and ZIF@ADH/NAD-MSN/LDH were determined by measuring N_2_ adsorption- desorption. The experimental results are shown in Table S1 and Figure S2. From Figure S2A,B, the average pore size of ZIF-8 complex was 17–22 nm, while the average pore size of ADH, LDH and coenzyme polymer was reduced to 3–4 nm after ZIF-8 embedding. The N_2_ adsorption-desorption curve of MSN and MSN-COOH were Langmuir IV type based on the characteristics and trend of the curve according to Figure S2C. It was the characteristic curve of a typical mesoporous material. As shown in Figure S2D, the N_2_ adsorption-desorption curve of ZIF-8 showed Langmuir IV type as well, while the N_2_ adsorption-desorption curve of ZIF@ADH/NAD-MSN/LDH was III type. From Table S1, the specific surface area of blank ZIF-8 material was 1298 m^2^/g, which decreased to 1118 m^2^/g when the enzyme and coenzyme (ADH, ADH, and MSN-NAD^+^) were introduced. It indicated that the enzyme and coenzyme polymers had been introduced into ZIF-8 materials. Although the enzyme was immobilized by in-situ synthesis, ZIF@ADH/NAD-MSN/LDH still had about 3 nm pores for the mass transfer of substrates and intermediate products. In summary, the core–shell structure polymer synthesized in situ was beneficial to the transformation of coenzymes in the two states, thereby improving the efficiency of the enzyme cascade reaction [54].

### 3.2. Evaluation of the Efficiency of ZIF@ADH/NAD-MSN/LDH Cascade Reaction in Nanoreactor

**Figure 4:**
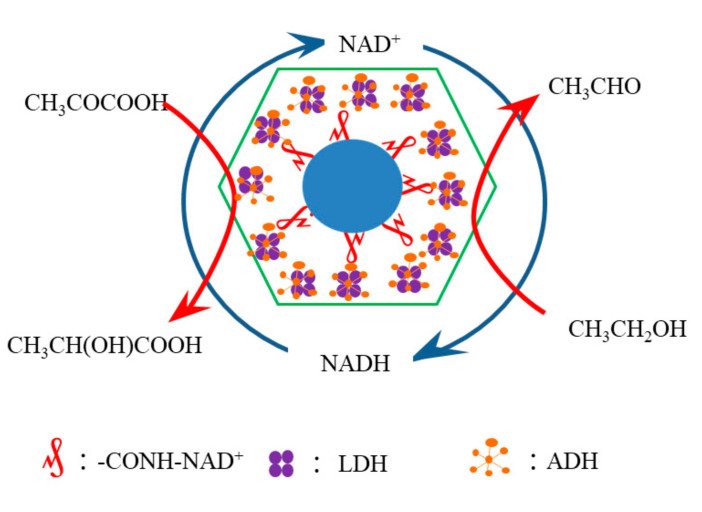
Flow chart of the reaction about the ZIF@ADH/NAD-MSN/LDH. ADH: Alcohol dehydrogenase; LDH: Lactate dehydrogenase; NAD^+^: Nicotinamide adenine dinucleotide; ZIF@ADH/NAD-MSN/LDH: Core–shell nanocomposites prepared by ZIF-8 immobilized ADH, LDH, and MSN-NAD.

In our research, the schematic diagram of the experimental mechanism is shown in Figure 4. This experiment was based on the following principles: the coenzyme was immobilized on the nano silica carrier by silane APTES modification and carboxylation modification. Under the action of accelerant, carboxyl group combined with inactive site of coenzyme to realize the immobilization of coenzyme and form MSN-NAD^+^. Then ADH, MSN-NAD^+^, and LDH were synthesized by in-situ polymerization in aqueous solution ZIF@ADH/NAD-MSN/LDH. ADH was a zinc-containing metalloenzyme with a wide range of substrate specificity. ADH used nicotinamide purine dinucleotide (NAD) as coenzyme to catalyze the reversible reaction between primary alcohols and aldehydes. ADH was a kind of NAD-dependent kinase, which had three subunits of LDHA, LDHB, and LDHC, and could form six kinds of tetramer isozymes. ADH was also a zinc-containing metalloenzyme. According to the difference of binding coenzymes, they were divided into two categories: NAD-dependent lactate dehydrogenases (nLDHs) and NAD-independent lactate dehydrogenases (iLDHs). NAD-dependent lactate dehydrogenases could use NADH as a cofactor to catalyze the reduction of pyruvate to lactic acid and oxidize NADH to NAD^+^. The catalysis of ADH and LDH made the continuous conversion of NAD^+^ and NADH to realize coenzyme redox regeneration and multi enzyme cascade [55]. 

Two coenzyme dependent enzymes were co-immobilized in the core–shell nano microenvironment defined by ZIF-8 and MSN-NAD^+^. With the co-immobilization, the physical distance between the co-immobilized enzymes was reduced to realize the cascade reaction of multiple enzymes, which was consistent with the report in the literature [56]. In the limited nanostructures, H^+^ and NADH produced by ADH in the process of ethanol conversion could quickly reach the LDH in the system, participate in the conversion of lactic acid and reduce the diffusion of substrate. NAD^+^ was involved in the catalysis of ethanol. The investment of expensive coenzyme was reduced by the cycle mechanism of coenzyme in the system. 

In order to proof the above reaction mechanism, the types of products produced by different components of ZIF-8 immobilized products with the addition of different reactants have been investigated. The experimental results are displayed in Table 1. It showed that the formation of lactic acid could not be catalyzed with only one enzyme or one reaction substrate. The coenzyme circulation and multi-enzyme cascade reaction could be realized only when coenzyme, ADH, LDH, ethanol and pyruvate existed at the same time. The multi-enzyme cascade reaction was divided into two steps. The first was the reaction between ADH and NAD to catalyze ethanol to produce acetaldehyde, H^+^ and NADH. Then the generated NADH and H^+^ participated in the reaction of LDH to catalyze the synthesis of lactic acid from pyruvate. 

**Figure 5:**
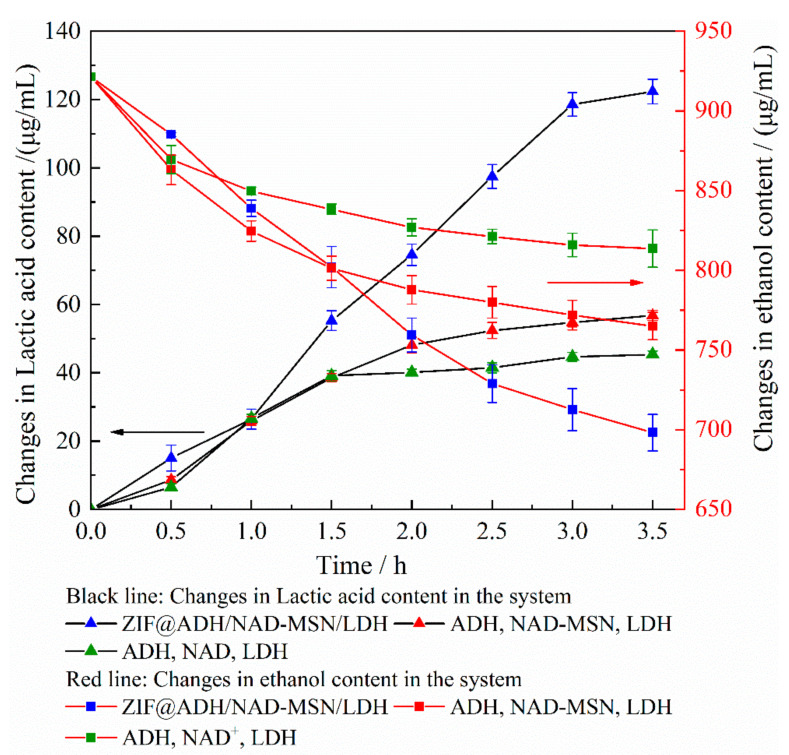
Changes in the content of lactic acid and ethanol in the system over time. ADH: Alcohol dehydrogenase; LDH: Lactate dehydrogenase; NAD^+^: Nicotinamide adenine dinucleotide; ZIF@ADH/NAD-MSN/LDH: Core–shell nanocomposites prepared by ZIF-8 immobilized ADH, LDH and MSN-NAD; MSN-NAD: Mesoporous silica nanoparticles immobilized nicotinamide adenine dinucleotide. The black line with blue triangles represented the amount of lactic acid synthesized in the ZIF@ADH/NAD-MSN/LDH experimental group. The black line with a red triangle represented the amount of lactic acid synthesized in the ADH, NAD-MSN, and LDH experimental groups. The black line with green triangles represented the amount of lactic acid synthesized in the ADH, NAD^+^, and LDH experimental groups. The red line with blue squares represented the amount of ethanol remaining in the ZIF@ADH/NAD-MSN/LDH experimental group. The red line with red squares represented the amount of ethanol remaining in the ADH, NAD-MSN, and LDH experimental groups. The red line with green squares represented the amount of ethanol remaining in the ADH, NAD^+^, and LDH experimental groups. Each experimental group was repeated three times.

Based on the above experimental principles, the relationship between the conversion of lactic acid and ethanol consumption in three reaction systems were tested. The experimental results are shown in Figure 5. It could be found that in the three reaction systems, the first step was the conversion of ethanol, and the generated NADH initiated the conversion of lactic acid. The concentration of lactic acid increased linearly from the first three hours of ZIF@ADH/NAD-MSN/LDH. After three hours, the conversion to ethanol and pyruvate tended to end. The concentration of lactic acid could reach 124.605 μg/mL and the conversion rate of pyruvate could reach more than 70%. The rapid conversion of NAD^+^ to NADH was the reason why the lactic acid yield of ZIF@ADH/NAD-MSN/LDH was higher than that of the other two experimental groups [57]. The concentration of lactic acid about the other two control groups were 58.850 μg/mL and 46.235 μg/mL, respectively. The conversion of lactic acid in these two control groups tends to end in about 2 h. The high production rate of ZIF@ADH/NAD-MSN/LDH was also related to the activity of immobilized enzyme. The consumption of pyruvate was determined by spectrophotometry and the activity of immobilized enzyme was 1.72 times higher than that of free enzyme. The conclusion was also consistent with the theory that microporous or macroporous materials could reduce substrate diffusion and improve the efficiency of product synthesis [58]. 

The reason why the lactic acid synthesis rate of the experimental group not higher than that of the control group at the beginning may be the inhibitory effect of acetaldehyde produced in the system on enzyme activity [59]. As the experiment progressed, acetaldehyde catalyzed by ethanol was dispersed out of ZIF@ADH/NAD-MSN/LDH through the pore structure of the material, and its impact on the enzyme was reduced. The diffusion position of the substrate in the reaction system was related to the conversion rate of the coenzyme. The farther the distance between the substrate diffusion position and the coenzyme, the worse the coenzyme regeneration ability, which further affected the synthesis of the reaction.

The results confirmed the feasibility of the catalytic cascade between two coenzyme-dependent enzymes with coenzyme polymer as the electronic medium in rigid NMOF particles. The enhancement of lactic acid conversion efficiency was related to the increased substrate concentration near the enzyme [60,61]. Therefore, the strategy of assembling two coenzyme-dependent enzymes into the same porous carrier was feasible to improve the cascade of multiple enzymes. Because the substrate and cofactors did not have to diffuse out of the porous material. This increased their concentration around the enzyme unit that forms the catalytic cascade [62]. In addition, the microenvironment created by the immobilized carrier ZIF-8 may also help increase the affinity of the substrate and thus increased the conversion efficiency of lactic acid [53,63]. Probably due to the inhibitory effect of the product acetaldehyde on the enzyme activity of ADH and LDH, the lactic acid cannot be completely converted [64]. How to separate the product of the system in time to reduce the impact on the reaction system should be paid attention to in the follow-up research.

### 3.3. Study on the Reusability and Stability of ZIF@ADH/NAD-MSN/LDH

**Figure 6:**
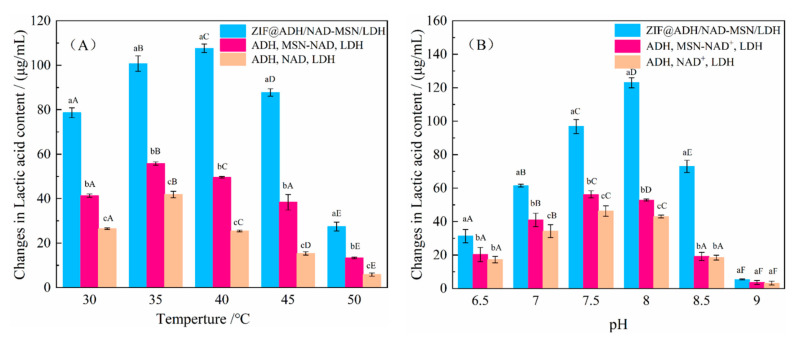
Changes in the content of lactic acid in the system under different conditions. ADH: Alcohol dehydrogenase; LDH: Lactate dehydrogenase; NAD^+^: Nicotinamide adenine dinucleotide; ZIF@ADH/NAD-MSN/LDH: Core–shell nanocomposites prepared by ZIF-8 immobilized ADH, LDH, and MSN-NAD; MSN-NAD: Mesoporous silica nanoparticles immobilized nicotinamide adenine dinucleotide. (**A**) Lactic acid synthesis concentration of three experimental groups within 3 h at different temperatures. The same lowercase letters indicated that there was no significant difference in the concentration of lactic acid synthesis in the three experimental groups at the same temperature. Different lowercase letters indicated that there was significant difference in the lactic acid synthesis concentration of the three experimental groups at the same temperature. The same capital letters represent that there was no significant difference in the concentration of lactic acid synthesis in the same experimental group at different temperatures. Different capital letters indicated that the lactic acid synthesis concentration of the same experimental group at different temperatures was significantly different. (**B**) Lactic acid synthesis concentration of three experimental groups in 3 h at different pH. The same lowercase letters indicated that there was no significant difference in the concentration of lactic acid synthesis in the three experimental groups at the same pH. Different lowercase letters indicated that there was significant difference in the lactic acid synthesis concentration of the three experimental groups at the same pH. The same capital letters represented that there was no significant difference in the concentration of lactic acid synthesis in the same experimental group at different pH. Different capital letters indicated that there was significant difference in lactic acid synthesis concentration in the same experimental group under different pH conditions.

The catalytic reaction rate of enzyme was related to temperature and pH value. When the reaction conditions were too extreme, the catalytic efficiency of the enzyme will be greatly reduced [65]. In this study, the production rate of lactic acid was used as an indicator, and the stability of the co-immobilized enzyme was measured and compared with the free enzyme. As shown in Figure 6A, the lactic acid production rate of co-immobilized enzyme significantly increased at 35–45 °C, with the optimum temperature at 40 °C. Moreover, the conversion of lactic acid was 2.82 times higher than that of free enzyme. Compared with another two groups, ZIF@ADH/NAD-MSN/LDH showed higher thermostability, which might be attributed to the enhanced rigidity of the protein secondary structure [7]. Enzyme catalytic reactions are easily affected by thermal inactivation of enzyme proteins. After exceeding the optimal reaction temperature, the concentration of lactic acid in the three experiments all decreased as the temperature increased. However, the production rate was still higher than that of the two control groups. It indicated the co-immobilized enzyme could be used in a wider temperature range.

It can be seen from Figure 6B that in the pH range of 7–8, the three experimental groups maintained high lactic acid production rate. There was no significant difference between ZIF@ADH/NAD-MSN/LDH and the other two enzyme treatments under the experimental conditions of pH = 6.5, 8.5, 9 (*p* > 0.05). The optimal pH value of ZIF@ADH/NAD-MSN/LDH for catalyzed synthesis of lactic acid was 8. While lactic acid could hardly be produced at pH 9. It was inclined to convert lactic acid to pyruvate from lactate dehydrogenase at this pH value. This reaction required a large amount of lactic acid substrate and NAD^+^. The system could not satisfy this condition, resulting in only a small amount of lactic acid produced in the reaction system. The results showed that under the protection of the MOF shell, the extreme pH and high temperature tolerances of the embedded enzyme were significantly enhanced, which was consistent with the literatures [66,67]. The ZIF-8 shell created an ideal micro-environment for the expression of the enzyme activity [68].

From a practical and economic point of view, the storage stability and reusability of the enzyme were the keys to its wide application. Therefore, the construction of a multi-enzyme cascade reaction system with the high storage stability and reusability was extremely important for practical applications. 

**Figure 7:**
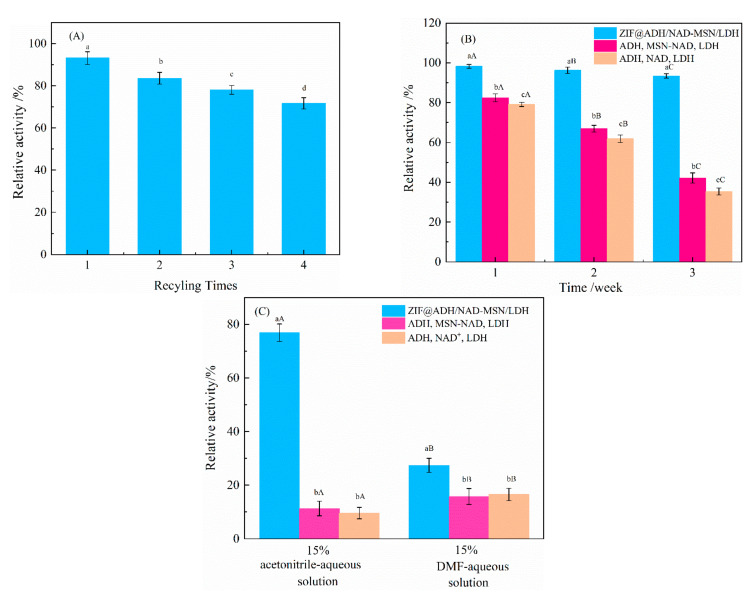
Reusability and stability. ADH: Alcohol dehydrogenase; LDH: Lactate dehydrogenase; NAD^+^: Nicotinamide adenine dinucleotide; ZIF@ADH/NAD-MSN/LDH: Core–shell nanocomposites prepared by ZIF-8 immobilized ADH, LDH and MSN-NAD; MSN-NAD: Mesoporous silica nanoparticles immobilized nicotinamide adenine dinucleotide. (**A**) The relative recovery rate of ZIF@ADH/NAD-polymer/LDH under different experiment times, (**B**) The relative activity of three experimental groups in PBS for three weeks at 4 °C. The same lowercase letters mean that there was no significant difference in enzyme activity between different experimental groups at the same time. Different lowercase letters represented significant differences in enzyme activity in different experimental groups The same capital letter means that there was no significant difference in the enzyme activity of the same experimental group at different times. Different capital letters indicate that the enzyme activity of the same experimental group at different times was significantly different. (**C**) The relative activity of the three experimental groups after incubating in 15% acetonitrile and DMF solution for 1 h. The same lowercase letters represent that there was no significant difference in the enzyme activity of different experimental groups treated in the same organic solution for the same time. Different lowercase letters indicated that there were significant differences in the enzyme activity of different experimental groups treated in the same organic solution. The same capital letters indicate that there was no significant difference in the enzyme activity of the same experimental group after the treatment with the two organic solutions. Different capital letters indicate that the enzyme activity of the same experimental group after the treatment with two organic solutions was significantly different. The experimental results were repeated three times.

The experimental results are shown in Figure 7A. The first relative recovery was 93.16%. After four reuses, the relative recovery could still reach to ~71.67%, indicating the good stability of the polymer. The loss of enzyme activities may occur as follows: (1) The centrifugal recovery of ZIF@ADH/NAD-MSN/LDH resulted in enzyme leakage and reduced enzyme activity [69]. (2) The acetaldehyde catalyzed by ethanol would affect the stability and catalytic effect of the enzyme in the circulation system. (3) The efficiency drops majorly resulted from the inevitable polymer weight loss in the separation from the reacting suspension [69]. It can be seen from Figure 7B that as the storage time increased, the enzyme activity under the system showed a gradual decline. However, the enzyme activity of ZIF@ADH/NAD-MSN/LDH remained at the initial 93.45% after three weeks, with almost no activity loss. The other two groups of free enzymes lost about 60% and 65% of their activity after three weeks. These results highlighted the excellent protective effect of MOF on enzyme.

The stability of the three experimental groups in two organic solvents is shown in Figure 7C. After incubating ZIF@ADH/NAD-MSN/LDH in 15% acetonitrile-aqueous solution and DMF for 1 h, the activity was retained at 76.89% (acetonitrile-aqueous solution) and 27.42% (DMF). The explanation for the difference in relative activity may be the size of the solvent molecule. DMF was a larger molecule than acetonitrile. Therefore, acetonitrile molecules could enter the pores of ZIF-8 and reach the encapsulated enzyme, thus destroying the enzyme activity. The smaller difference in enzyme activity between free enzyme and ZIF@ADH/NAD-MSN/LDH in 15% acetonitrile-aqueous solution 1 h also confirmed the above possibility. However, compared with free enzymes, ZIF@ADH/NAD-MSN/LDH showed better tolerance to organic solvents and better stability to organic solvents. The barrier formed by the ZIF-8 channel could protect the enzyme by preventing some macromolecular substances from approaching the enzyme molecules.

## 4. Conclusions

In this paper, ADH and LDH were co-immobilized in situ in the area defined by ZIF-8. The catalysis of ethanol and pyruvate triggered the cyclic regeneration of coenzymes to further realized the rapid synthesis of lactic acid. Compared with the lactic acid synthesis system catalyzed by biological enzymes, ZIF@ADH/NAD-MSN/LDH does not require additional coenzymes. From a practical point of view, ZIF@ADH/NAD-MSN/LDH was more economical. This multi-enzyme cascade reaction strategy could also be applied to other in vitro artificially prepared multi-enzyme reaction systems. 

## Figures and Tables

**Table 1:** Conditions of the multi-enzyme cascade reaction and the types of products.

Reaction Conditions		The Types of Products
ZIF-8 Immobilized Components ^1^	Reactant ^2^	
ADH ^3^	C_2_H_5_OH CH_3_COCOOH	No reaction
LDH ^4^	C_2_H_5_OH CH_3_COCOOH	No reaction
LDH MSN-NAD ^5^	C_2_H_5_OH CH_3_COCOOH	No reaction
ADH MSN-NAD^+^	C_2_H_5_OH CH_3_COCOOH	CH_3_CHO
ADH LDH	C_2_H_5_OH CH_3_COCOOH	No reaction
ADH LDH MSN-NAD^+^	C_2_H_5_OH	CH_3_CHO
ADH LDH MSN-NAD^+^	CH_3_COCOOH	No reaction
ADH LDH MSN-NAD^+^	C_2_H_5_OH CH_3_COCOOH	CH_3_CHO CH_3_CH(OH)COOH

^1^ The total mass of enzymes in ZIF-8 was 1 mg, and the amount of MSN-NAD^+^ was 1 mg. The preparation method of different ZIF-8 system was consistent with 2.3; ^2^ In the PB solution with pH = 7.4, added one or two substrates to study the key factors of the multi-enzyme cascade. The addition amount of the two substrates and polymers was consistent with 2.5; ^3^ Alcohol dehydrogenase; ^4^ Lactate dehydrogenase; ^5^ Mesoporous silica nanoparticles immobilized nicotinamide adenine dinucleotide.

## Data Availability

The data presented in this study are available within this article and its Supplementary Materials.

## Supplementary Materials

The following are available online at https://www.mdpi.com/article/10.3390/nano11092171/s1, Figure S1: Schematic diagram of MSN-NAD preparation process, Figure S2: The pore volume and N_2_ adsorption–desorption isotherms for MSN, MSN-COOH, ZIF-8 and ZIF@ADH/NAD-MSN/LDH, Table S1: Results of the specific surface area of ZIF-8, MSN, MSN-COOH and ZIF@ADH/NAD-MSN /LDH.

## References

[1] Wichmann R., Wandrey C., Bückmann A.F., Kula M.R. (1981). Continuous enzymatic transformation in an enzyme membrane reactor with simultaneous NAD(H) regeneration. Biotechnol. Bioeng..

[2] Gasparrini M., Sorci L., Raffaelli N. (2021). Enzymology of extracellular NAD metabolism. Cell. Mol. Life Sci..

[3] Singh C., Kim T.W., Yadav R.K., Kumar K., Yadav B.C. (2021). Anthracene-based g-C_3_N_4_ photocatalyst for regeneration of NAD(P)H and sulfide oxidation based on Z-scheme nature. Int. J. Energy Res..

[4] Anne A., Bourdillon C., Daninos S., Moiroux J. (1999). Can the combination of electrochemical regeneration of NAD^+^, selectivity of L-α-amino-acid dehydrogenase, and reductive amination of α- keto-acid be applied to the inversion of configuration of a L-α-amino-acid?. Biotechnol. Bioeng..

[5] Chen Q., Yu S., Myung N., Chen W. (2017). DNA-guided assembly of a five-component enzyme cascade for enhanced conversion of cellulose to gluconic acid and H_2_O_2_. J. Biotechnol..

[6] Xu L., Wang L.C., Su B.M., Xu X.Q., Lin J. (2020). Multi-enzyme cascade for improving β-hydroxy-α-amino acids production by engineering L-threonine transaldolase and combining acetaldehyde elimination system. Bioresour. Technol..

[7] Yu X., Zhang Z., Li J., Su Y., Gao M., Jin T., Chen G. (2021). Co-immobilization of multi-enzyme on reversibly soluble polymers in cascade catalysis for the one-pot conversion of gluconic acid from corn straw. Bioresour. Technol..

[8] Findrik Z., Vasić-Rački D. (2009). Overview on reactions with multi-enzyme systems. Chem. Biochem. Eng. Q..

[9] Faber K. (2009). Multi-Step Enzyme Catalysis: Biotransformations and Chemoenzymatic Synthesis. Edited by Eduardo García-Junceda. ChemBioChem.

[10] Wilner O.I., Weizmann Y., Gill R., Lioubashevski O., Freeman R., Willner I. (2009). Enzyme cascades activated on topologically programmed DNA scaffolds. Nat. Nanotechnol..

[11] Vong T., Schoffelen S., van Dongen S.F.M., van Beek T.A., Zuilhof H., van Hest J.C.M. (2011). A DNA-based strategy for dynamic positional enzyme immobilization inside fused silica microchannels. Chem. Sci..

[12] Xin L., Zhou C., Yang Z., Liu D. (2013). Regulation of an enzyme cascade reaction by a DNA machine. Small.

[13] Zhang F., Jiang S., Wu S., Li Y., Mao C., Liu Y., Yan H. (2015). Complex wireframe DNA origami nanostructures with multi-arm junction vertices. Nat. Nanotechnol..

[14] Talekar S., Pandharbale A., Ladole M., Nadar S., Mulla M., Japhalekar K., Pattankude K., Arage D. (2013). Carrier free co-immobilization of alpha amylase, glucoamylase and pullulanase as combined cross-linked enzyme aggregates (combi-cleas): A tri-enzyme biocatalyst with one pot starch hydrolytic activity. Bioresour. Technol..

[15] Patterson D.P., Schwarz B., Waters R.S., Gedeon T., Douglas T. (2014). Encapsulation of an enzyme cascade within the bacteriophage P22 virus-like particle. ACS Chem. Biol..

[16] Pescador P., Katakis I., Toca-Herrera J.L., Donath E. (2008). Efficiency of a bienzyme sequential reaction system immobilized on polyelectrolyte multilayer-coated colloids. Langmuir.

[17] Fontes C.M.G.A., Gilbert H.J. (2010). Cellulosomes: Highly efficient nanomachines designed to deconstruct plant cell wall complex carbohydrates. Annu. Rev. Biochem..

[18] Oroz-Guinea I., García-Junceda E. (2013). Enzyme catalysed tandem reactions. Curr. Opin. Chem. Biol..

[19] Delaittre G., Reynhout I.C., Cornelissen J.J.L.M., Nolte R.J.M. (2009). Cascade reactions in an all-enzyme nanoreactor. Chem. Eur. J..

[20] Kuiper S.M., Nallani M., Vriezema D.M., Cornelissen J.J.L.M., Van Hest J.C.M., Nolte R.J.M., Rowan A.E. (2008). Enzymes containing porous polymersomes as nano reaction vessels for cascade reactions. Org. Biomol. Chem..

[21] Torabi S.F., Khajeh K., Ghasempur S., Ghaemi N., Siadat S.O.R. (2007). Covalent attachment of cholesterol oxidase and horseradish peroxidase on perlite through silanization: Activity, stability and co-immobilization. J. Biotechnol..

[22] Gan J.S., Bagheri A.R., Aramesh N., Gul I., Franco M., Almulaiky Y.Q., Bilal M. (2021). Covalent organic frameworks as emerging host platforms for enzyme immobilization and robust biocatalysis—A review. Int. J. Biol. Macromol..

[23] Reis C.L.B., de Sousa E.Y.A., de França Serpa J., Oliveira R.C., Dos Santos J.C.S. (2019). Design of immobilized enzyme biocatalysts: Drawbacks and opportunities. Quim. Nova.

[24] Aggarwal S., Chakravarty A., Ikram S. (2021). A comprehensive review on incredible renewable carriers as promising platforms for enzyme immobilization & thereof strategies. Int. J. Biol. Macromol..

[25] Horcajada P., Gref R., Baati T., Allan P.K., Maurin G., Couvreur P., Férey G., Morris R.E., Serre C. (2012). Metal-organic frameworks in biomedicine. Chem. Rev..

[26] Chen W.H., Yu X., Liao W.C., Sohn Y.S., Cecconello A., Kozell A., Nechushtai R., Willner I. (2017). ATP-Responsive Aptamer-Based Metal–Organic Framework Nanoparticles (NMOFs) for the Controlled Release of Loads and Drugs. Adv. Funct. Mater..

[27] Chen W.H., Yu X., Cecconello A., Sohn Y.S., Nechushtai R., Willner I. (2017). Stimuli-responsive nucleic acid-functionalized metal-organic framework nanoparticles using pH- and metal-ion-dependent DNAzymes as locks. Chem. Sci..

[28] Chen W.H., Liao W.C., Sohn Y.S., Fadeev M., Cecconello A., Nechushtai R., Willner I. (2018). Stimuli-Responsive Nucleic Acid-Based Polyacrylamide Hydrogel-Coated Metal–Organic Framework Nanoparticles for Controlled Drug Release. Adv. Funct. Mater..

[29] Chouyyok W., Panpranot J., Thanachayanant C., Prichanont S. (2009). Effects of pH and pore characters of mesoporous silicas on horseradish peroxidase immobilization. J. Mol. Catal. B Enzym..

[30] Jesionowski T., Zdarta J., Krajewska B. (2014). Enzyme immobilization by adsorption: A review. Adsorption.

[31] Homouz D., Stagg L., Wittung-Stafshede P., Cheung M.S. (2009). Macromolecular crowding modulates folding mechanism of α/β protein apoflavodoxin. Biophys. J..

[32] Wu X., Yang C., Ge J. (2017). Green synthesis of enzyme/metal-organic framework composites with high stability in protein denaturing solvents. Bioresour. Bioprocess..

[33] Cao X., Ni Y., Zhang A., Xu S., Chen K., Ouyang P. (2017). Encapsulation of enzymes in metal ion-surfactant nanocomposites for catalysis in highly polar solvents. Chem. Commun..

[34] Wang K., Ren H., Li N., Tan X., Dang F. (2018). Ratiometric fluorescence sensor based on cholesterol oxidase-functionalized mesoporous silica nanoparticle@ZIF-8 core-shell nanocomposites for detection of cholesterol. Talanta.

[35] Liu W., Zhang S., Wang P. (2009). Nanoparticle-supported multi-enzyme biocatalysis with in situ cofactor regeneration. J. Biotechnol..

[36] Yang S., Song S., Han K., Wu X., Chen L., Hu Y., Wang J., Liu B. (2019). Characterization, in vitro evaluation and comparative study on the cellular internalization of mesoporous silica nanoparticle-supported lipid bilayers. Microporous Mesoporous Mater..

[37] Shen Y., Zhang Y., Zhang X., Zhou X., Teng X., Yan M., Bi H. (2015). Horseradish peroxidase-immobilized magnetic mesoporous silica nanoparticles as a potential candidate to eliminate intracellular reactive oxygen species. Nanoscale.

[38] Bradford M.M. (1976). A rapid and sensitive method for the quantitation of microgram quantities of protein utilizing the principle of protein-dye binding. Anal. Biochem..

[39] Wang L., Sha Y., Wu D., Wei Q., Chen D., Yang S., Jia F., Yuan Q., Han X., Wang J. (2020). Surfactant induces ROS-mediated cell membrane permeabilization for the enhancement of mannatide production. Process Biochem..

[40] Niu B., Wu D., Wang J., Wang L., Zhang W. (2020). Salt-sealing-pyrolysis derived Ag/ZnO@C hollow structures towards efficient photo-oxidation of organic dye and water-born bacteria. Appl. Surf. Sci..

[41] Park K.S., Ni Z., Côté A.P., Choi J.Y., Huang R., Uribe-Romo F.J., Chae H.K., O’Keeffe M., Yaghi O.M. (2006). Exceptional chemical and thermal stability of zeolitic imidazolate frameworks. Proc. Natl. Acad. Sci. USA.

[42] Yang P., Quan Z., Li C., Kang X., Lian H., Lin J. (2008). Bioactive, luminescent and mesoporous europium-doped hydroxyapatite as a drug carrier. Biomaterials.

[43] Sun Y., Sun Y.L., Wang L., Ma J., Yang Y.W., Gao H. (2014). Nanoassembles constructed from mesoporous silica nanoparticles and surface-coated multilayer polyelectrolytes for controlled drug delivery. Microporous Mesoporous Mater..

[44] Qin Y., Han X., Li Y., Han A., Liu W., Xu H., Liu J. (2020). Hollow Mesoporous Metal-Organic Frameworks with Enhanced Diffusion for Highly Efficient Catalysis. ACS Catal..

[45] Cui J., Wang L., Han Y., Liu W., Li Z., Guo Z., Hu Y., Chang Z., Yuan Q., Wang J. (2018). ZnO nano-cages derived from ZIF-8 with enhanced anti mycobacterium-tuberculosis activities. J. Alloys Compd..

[46] Sarrouh B. (2012). Up-To-Date Insight on Industrial Enzymes Applications and Global Market. J. Bioprocess. Biotech..

[47] Sheldon R.A. (2007). Enzyme immobilization: The quest for optimum performance. Adv. Synth. Catal..

[48] Song Y., Hu D., Liu F., Chen S., Wang L. (2015). Fabrication of fluorescent SiO_2_@zeolitic imidazolate framework-8 nanosensor for Cu2+ detection. Analyst.

[49] Son J., Lee H.J., Oh M. (2013). Systematic formation of multilayered core-shell microspheres through the multistep growth of coordination polymers. Chem. Eur. J..

[50] Wang G., Sun T., Sun Z., Hu X. (2020). Preparation of copper based metal organic framework materials and its effective adsorptive removal of ceftazidime from aqueous solutions. Appl. Surf. Sci..

[51] Chen W.H., Vázquez-González M., Zoabi A., Abu-Reziq R., Willner I. (2018). Biocatalytic cascades driven by enzymes encapsulated in metal–organic framework nanoparticles. Nat. Catal..

[52] Song J., He W., Shen H., Zhou Z., Li M., Su P., Yang Y. (2019). Construction of multiple enzyme metal–organic frameworks biocatalyst via DNA scaffold: A promising strategy for enzyme encapsulation. Chem. Eng. J..

[53] Lyu F., Zhang Y., Zare R.N., Ge J., Liu Z. (2014). One-pot synthesis of protein-embedded metal-organic frameworks with enhanced biological activities. Nano Lett..

[54] Zhao M., Li Y., Ma X., Xia M., Zhang Y. (2019). Adsorption of cholesterol oxidase and entrapment of horseradish peroxidase in metal-organic frameworks for the colorimetric biosensing of cholesterol. Talanta.

[55] Hollmann F., Hofstetter K., Schmid A. (2006). Non-enzymatic regeneration of nicotinamide and flavin cofactors for monooxygenase catalysis. Trends Biotechnol..

[56] Han P., Zhou X., You C. (2020). Efficient Multi-Enzymes Immobilized on Porous Microspheres for Producing Inositol From Starch. Front. Bioeng. Biotechnol..

[57] Eggert M.W., Byrne M.E., Chambers R.P. (2011). Impact of high pyruvate concentration on kinetics of rabbit muscle lactate dehydrogenase. Appl. Biochem. Biotechnol..

[58] Guo Y., Feng L., Wu C., Wang X., Zhang X. (2019). Synthesis of 3D-Ordered Macro/Microporous Yolk-Shelled Nanoreactor with Spatially Separated Functionalities for Cascade Reaction. ACS Appl. Mater. Interfaces.

[59] Lopez-Gallego F., Batencor L., Hidalgo A., Mateo C., Fernandez-Lafuente R., Guisan J.M. (2005). One-pot conversion of cephalosporin C to 7-aminocephalosporanic acid in the absence of hydrogen peroxide. Adv. Synth. Catal..

[60] Garcia J., Zhang Y., Taylor H., Cespedes O., Webb M.E., Zhou D. (2011). Multilayer enzyme-coupled magnetic nanoparticles as efficient, reusable biocatalysts and biosensors. Nanoscale.

[61] Chen S., Wen L., Svec F., Tan T., Lv Y. (2017). Magnetic metal-organic frameworks as scaffolds for spatial co-location and positional assembly of multi-enzyme systems enabling enhanced cascade biocatalysis. RSC Adv..

[62] Rocha-Martín J., de Las Rivas B., Muñoz R., Guisán J.M., López-Gallego F. (2012). Rational co-immobilization of bi-enzyme cascades on porous supports and their applications in bio-redox reactions with insitu recycling of soluble cofactors. ChemCatChem.

[63] Kim J., Grate J.W., Wang P. (2008). Nanobiocatalysis and its potential applications. Trends Biotechnol..

[64] Knedel T.O., Ricklefs E., Schlüsener C., Urlacher V.B., Janiak C. (2019). Laccase Encapsulation in ZIF-8 Metal-Organic Framework Shows Stability Enhancement and Substrate Selectivity. ChemistryOpen.

[65] Long J., Pan T., Xie Z., Xu X., Jin Z. (2020). Co-immobilization of β-fructofuranosidase and glucose oxidase improves the stability of Bi-enzymes and the production of lactosucrose. LWT Food Sci. Technol..

[66] He H., Han H., Shi H., Tian Y., Sun F., Song Y., Li Q., Zhu G. (2016). Construction of Thermophilic Lipase-Embedded Metal-Organic Frameworks via Biomimetic Mineralization: A Biocatalyst for Ester Hydrolysis and Kinetic Resolution. ACS Appl. Mater. Interfaces.

[67] Wu X., Ge J., Yang C., Hou M., Liu Z. (2015). Facile synthesis of multiple enzyme-containing metal-organic frameworks in a biomolecule-friendly environment. Chem. Commun..

[68] Sannino F., Costantini A., Ruffo F., Aronne A., Venezia V., Califano V. (2020). Covalent immobilization of β-glucosidase into mesoporous silica nanoparticles from anhydrous acetone enhances its catalytic performance. Nanomaterials.

[69] Wang L., Ma C., Ru X., Guo Z., Wu D., Zhang S., Yu G., Hu Y., Wang J. (2015). Facile synthesis of ZnO hollow microspheres and their high performance in photocatalytic degradation and dye sensitized solar cells. J. Alloys Compd..

