# Supplementary material for: Preparation of ZIF@ADH/NAD-MSN/LDH Core Shell Nanocomposites for the Enhancement of Coenzyme Catalyzed Double Enzyme Cascade
Source: Nanomaterials (Basel). 2021 Aug 25;11(9):2171. doi: 10.3390/nano11092171 (PMC8464746; doi:10.3390/nano11092171)
Supplement: Supplementary file 1 [file nanomaterials-11-02171-s001.zip › nanomaterials-1323260-supplementary.pdf]

nanomaterials

Supplementary Materials

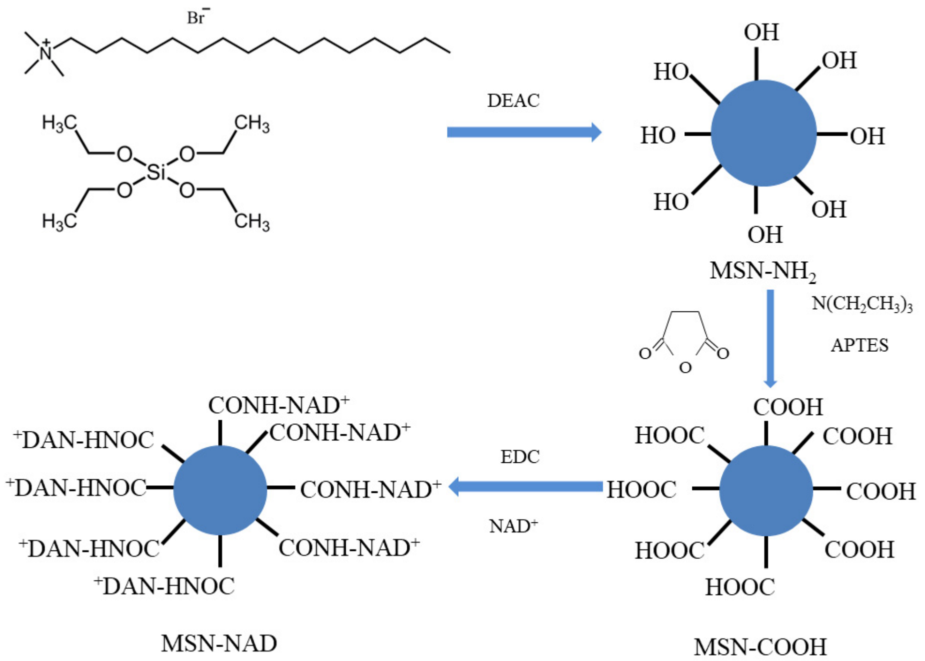

Figure S1. Schematic diagram of MSN-NAD preparation process. EDC: 1-(3-Dimethylaminopropyl)-3-ethylcar bodiimide hydrochloride; DEAC: Diethylene glycol mono-ethyl ether acetate; MSN-NH_2: Amino modified mesoporous silica nanoparticles; MSN-COOH: Carboxyl modified mesoporous silica nano particles; NAD^+: Nicotinamide adenine dinucleotide; MSN-NAD: Mesoporous silica nanoparticles immobilized nicotinamide adenine dinucleotide.

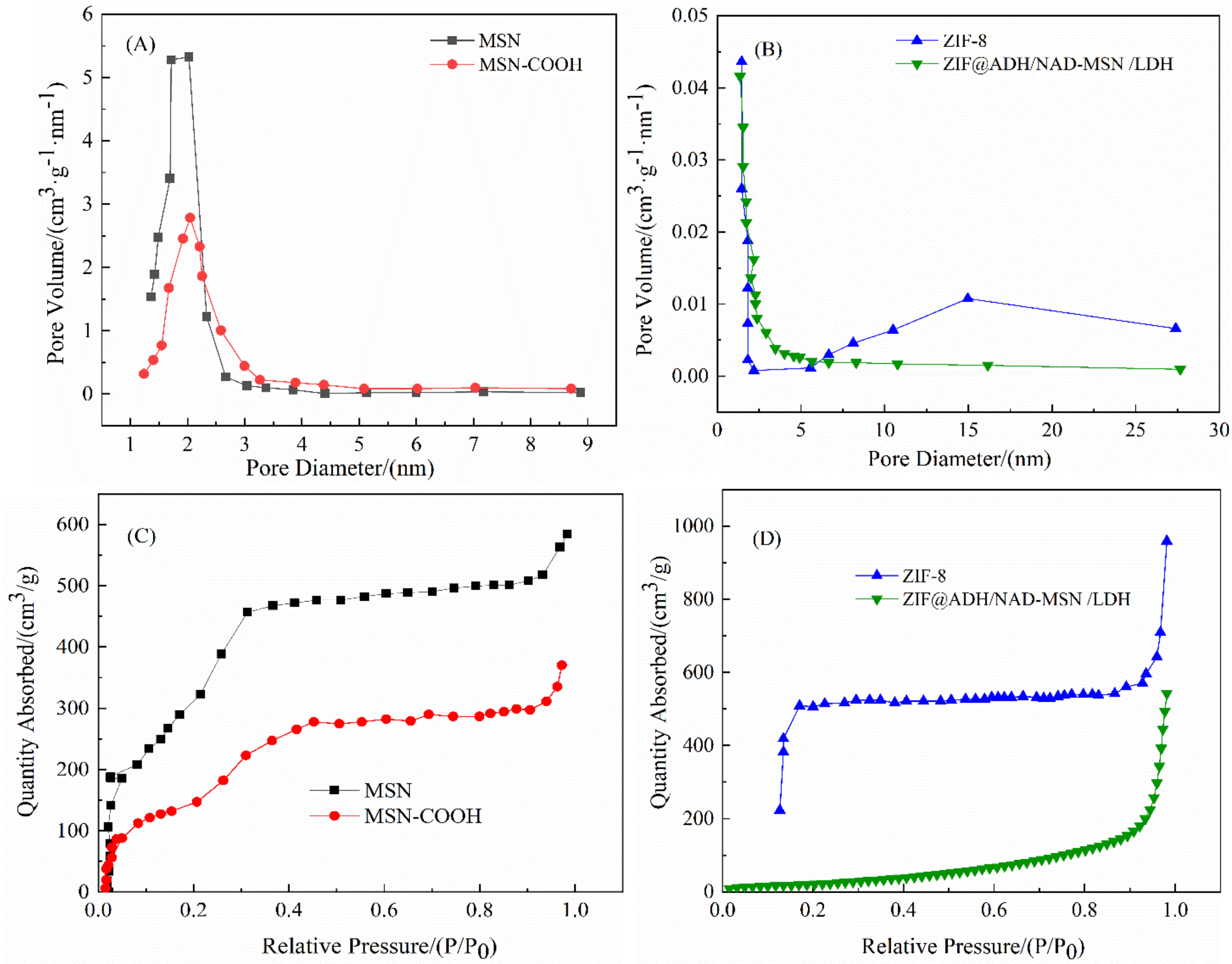

Figure S2. The pore volume and N_2 adsorption–desorption isotherms for MSN, MSN-COOH, ZIF-8 and ZIF@ADH/NAD-MSN/LDH. MSN: Mesoporous silica nanoparticles; MSN-COOH: Carboxyl modified mesoporous silica nanoparticles; ZIF-8: A representative material in the zeolitic imidazolate frame-works series; ZIF@ADH/NAD-MSN/LDH: Core-shell nanocomposites prepared by ZIF-8 immobilized ADH, LDH and MSN-NAD. (**A**) The change of pore volume before and after MSN carboxyl modification, (**B**) The change of pore volume before and after immobilization of ZIF-8 with multiple enzymes, (**C**) The change of specific surface area of MSN before and after carboxyl modification, (**D**) Changes of the specific surface area before and after the immobilization of multiple enzymes on ZIF-8.

Table S1. Results of the specific surface area of ZIF-8, MSN, MSN-COOH and ZIF@ADH/NAD-MSN /LDH.

Sample	Specific Surface Area(m^2/g)
MSN[1]	544.88
MSN-COOH[2]	502.33
ZIF-8[3]	1197.56
ZIF@ADH/NAD-MSN/LDH[4]	1045.75

[1] Mesoporous silica nanoparticles;

[2] Carboxyl modified mesoporous silica nanoparticles;

[3] A representative material in the zeolitic imidazolate frame-works series;

[4] Core-shell nanocomposites prepared by ZIF-8 immobilized ADH, LDH and MSN-NAD
